# Double plate fixation improves stiffness in a comminuted canine scapula fracture gap model compared to single plate fixation

**DOI:** 10.1111/vsu.70008

**Published:** 2025-10-02

**Authors:** Faolain M. Barrett, Josh D. Roth, Herman Feller, Jessica McCarthy

**Affiliations:** ^1^ Department of Surgical Sciences, School of Veterinary Medicine University of Wisconsin‐Madison Madison Wisconsin USA; ^2^ Department of Orthopedics and Rehabilitation University of Wisconsin‐Madison Madison Wisconsin USA; ^3^ Present address: Veterinary Health Center University of Missouri Columbia Missouri USA

## Abstract

**Objective:**

To determine whether a secondary plate on the caudolateral aspect of the scapula increases stiffness and reduces primary plate strain compared to a single plate along the cranial scapula spine in a comminuted fracture gap model.

**Study design:**

Ex vivo mechanical study.

**Sample population:**

A total of 14 paired canine scapulae.

**Methods:**

A comminuted fracture gap model was created. A 2.4 mm plate was secured along the cranial aspect of the scapula spine in 28 scapulae. A secondary 2.0 mm plate was secured on the caudolateral border of 14 scapulae. Scapula were sinusoidally loaded from −20 to −200 N for 7200 cycles at 2 Hz. The displacement was measured, and stiffness calculated. Digital image correlation calculated primary plate surface strain. A two‐way ANOVA assessed displacement and stiffness. Primary plate strain was assessed with a paired *t*‐test. Statistical significance was set at *p* < .05.

**Results:**

Mean displacement was higher in the single plate group, −0.81 mm (± 0.14) compared to the double plate group, −0.48 mm (± 0.08) (*p* < .0001). Mean stiffness was lower in the single plate group, 392.8 N/mm (± 13.72) compared to the double plate group, 563.7 N/mm (± 5.89) (*p* <.0001). There was no difference in primary plate surface strain between the two groups.

**Conclusion:**

Double plate fixation improved stiffness in a comminuted scapula fracture gap model compared to single plate fixation.

**Clinical significance:**

The placement of an additional plate placed on the caudolateral aspect of the scapula improves stiffness in comminuted scapula body fractures.

## 1 INTRODUCTION

Comminuted scapula body fractures are a relatively uncommon injury in dogs but can cause substantial morbidity. There is no evidence‐based clinical consensus on the optimum treatment of these complex fractures.^1^, ^2^ Conservative medical management is not recommended for comminuted fractures of the scapula body since the movement of the fragments can cause significant swelling and discomfort. The main segments tend to override with the fracture edges angulating laterally leading to pain, reduced function, and increased potential for reinjury.^1^ Plate fixation is often recommended, and with the wide availability of locking plate fixation, there is improved implant resistance to screw pull out.^3^ The greatest bone purchase is achieved if screws are placed at a 45° angle to the scapula spine.^4^ This allows secure placement of a single plate, which has been shown to be biomechanically strong enough for simple scapula body fractures,^5^ but may be inadequate for comminuted scapula fractures.

Comminuted long bone fractures can be treated with dual bone plate fixation to achieve sufficient construct stiffness to allow immediate weightbearing and bone healing to occur.^6^ The use of a single bone plate in comminuted long bone fractures can lead to implant failure through fatigue fracture or bending.^7^, ^8^, ^9^ Double plating of feline ilial fractures in vivo leads to significantly less implant failure and pelvic canal narrowing compared to a single lateral plate.^10^ In one study of scapula fractures, a dual‐plate approach did not show a biomechanical strength advantage in a simple osteotomy model.^5^ However, this study used two plates in close proximity on either side of the scapula spine, which may have lessened the overall biomechanical advantage of having two plates. The caudal edge of the scapula must resist high tensile forces from the triceps brachii (long head) muscle and teres minor distally and rhomboid and teres major muscle proximally.^11^ Placing the second plate closer to the caudal edge of the scapula may provide a biomechanical advantage in vivo by better resisting these forces and avoids the suprascapular nerve compared to adding a plate closer to the cranial edge of the scapula.

The aim of the present study was to determine whether the addition of a secondary plate on the caudolateral aspect of the scapula would increase construct stiffness and reduce primary plate strain compared to a single plate along the cranial scapula spine in a comminuted fracture gap model. We hypothesized that adding a secondary plate to the caudal lateral aspect of the scapula would increase construct stiffness, reduce displacement, and reduce primary plate strain.

## 2 MATERIALS AND METHODS

### 2.1 Specimens

Paired scapulae from 14 adult Beagles were procured from cadavers utilized in teaching anatomy at the University of Wisconsin‐Madison. Scapulae were stored frozen at −20°C, and testing was performed within 24 hours of thawing at room temperature. All soft tissues were removed from the scapula, and the bones were visually free of abnormalities. During specimen preparation and testing, each scapula was wrapped in saline‐soaked gauze and drying was mitigated by periodic spraying with a saline solution (0.9% NaCl). A custom three‐dimensional (3D)‐printed guide was used to perform transverse 15 mm ostectomies on all scapulae to create a gap representative of comminution at the junction of the proximal 2/3 and distal one third of the bone. In all 28 scapulae, a single, nine‐hole, 2.4 mm Arthrex OrthoLine locking compression plate (Naples, Florida) was secured along the cranial aspect of the scapula spine with the same combination of locking, cortical, and variable angle locking screws in each plate. Prior to placement, the most proximal screw hole was removed from the plate using bolt cutters to reduce it to an eight‐hole length to ensure proper fit on the scapula. For each pair, either the right or left was selected with a coin flip and a secondary, seven‐hole, 2.0 mm Arthrex OrthoLine locking compression plate was secured on the caudolateral border of the scapula using the same combination of locking, cortical, and variable angle screws in each plate. All repairs were performed by the same surgeon (JMc).

### 2.2 Specimen preparation

A 3D digital image correlation (3D DIC) was used to measure surface strain during testing.^12^, ^13^ A random speckled pattern was applied by hand to each specimen onto the implants spanning the ostectomy and onto the bone and implants 15 mm either side of the ostectomy, using an Ultra Fine Tip black marker (Sharpie) to measure the von Mises strain using 3D image correlation. Prior to potting, two wood screws were placed in the proximal aspect of each scapula on each side of the scapular spine to improve fixation within the potting material. Each scapula was placed in a custom 3D‐printed potting guide with jig to ensure each scapula was potted straight in the three anatomic planes. The proximal one‐quarter of the dorsal scapula was potted in fiberglass resin (Bondo, 3M, Minnesota).

The specimens were placed on a custom wedge in the testing apparatus so that the long axis of the scapular spine was oriented 20° from vertical and clamped in place on a translating table to position each scapula correctly as utilized in previous scapula ex vivo studies.^5^ A custom 3D‐printed actuator head mimicking the articular surface of the humerus was created to prevent the load applicator from slipping and was positioned against the glenoid cavity, perpendicular to the articular surface.

During loading, high‐definition video was recorded every 30 seconds using four high‐definition Raspberry Pi cameras with 1080 p resolution. The cameras were positioned so that at least two cameras captured the primary plate over the fracture gap. The Raspberry Pi cameras had a frame rate of 30 fps, and an LED light was used to signal the onset of loading to synchronize frames from multiple cameras. 3D DIC was performed using the open‐source MATLAB program MultiDIC. Regions of interest (ROIs) were established on the primary plate over the fracture gap to measure the surface strain.

All 28 samples underwent cyclic testing using a mechanical testing machine (858 Bionix; MTS Systems). Each scapula was sinusoidally loaded from −20 to −200 N for 7200 cycles at 2 Hz. This simulated a 15 kg dog walking a total of 1 hour to mimic postoperative activity. The calculation to determine load was derived from the study by McLaughlin and Roush in Greyhounds where the peak vertical force of the forelimb at 3 m/s was reported to be approximately 125% of bodyweight.^14^ A 15 kg crossbreed dog was observed walking at a natural pace and the number of steps counted for 5 min. The average steps per minute was calculated to give 120 steps/min therefore, to simulate 1 hour of walking 7200 cycles were used. For each scapula, the displacement was measured, and the stiffness and plate surface strain were calculated.

### 2.3 Statistical analysis

All statistical analysis was performed using GraphPad Prism (GraphPad Prism 9.4.1, Dotmatics, Boston, Massachusetts). A priori power analysis for sample size calculation was performed using data from a previous scapula ex vivo study.^5^ We estimated that 20 dogs in a paired study design would provide 80% power to detect a 25% increase in scapula stiffness using two plates compared to one, with *p* < .05, and 0.6 effect size. A sample size of *n* = 14 dogs was used due to design differences. A post hoc analysis of all acquired data showed an effect size of 0.99 for mean displacement, 0.87 for maximum displacement and 0.99 for stiffness. Displacement (mm), stiffness (N/mm), and strain (mm/mm) data were evaluated. The mean of the values was calculated every 30 cycles. Statistical analysis was performed on the means from the first 120 cycles, middle 120 cycles and last 120 cycles to give 12 data points for each specimen. Data were assessed for normality using the Shapiro–Wilk test and visual inspection of Q‐Q plots. Displacement and stiffness data exhibited heavy‐tailed distributions. Skewness and Kurtosis were assessed for each overall mean and median and it was determined that they appropriate to allow parametric testing. Interclass correlation coefficient was used to assess dog effect. Two‐way ANOVA was performed to assess the effects of specimen, treatment group (single vs. double plate) and cycle number. Interaction effects between group and cycle were also evaluated. When a significant main effect or interaction was identified, post hoc multiple comparisons were performed using Bonferri's test to control for type I error. A *p*‐value of <.05 was used to determine statistical significance with 95% confidence interval. Primary plate surface strain data was normally distributed, and statistical analysis was performed with a paired *t*‐test with statistical significance of *p* < .05.

## 3 RESULTS

In total, 28 scapulae were loaded from −20 to −200 N for 7200 cycles. For one specimen, the MTS stopped working due to technical issues at 1250 cycles and was therefore excluded from the study. There was no construct failure. Slippage of the actuator head on the glenoid occurred in three specimens, all in the double plate group, which were excluded from statistical analysis. Slippage occurred at variable time points during testing. This resulted in 13 single plate specimens and 11 double plate specimens for statistical analysis.

**FIGURE 1:**
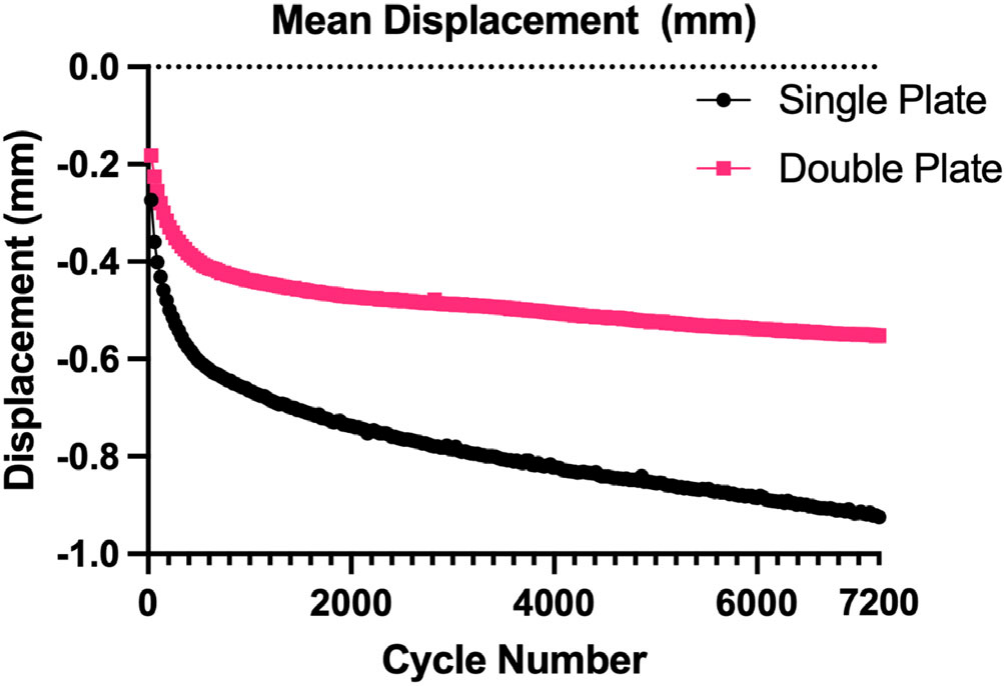
Mean displacement (mm) of all single and double plated scapula over 7200 cycles.

**FIGURE 2:**
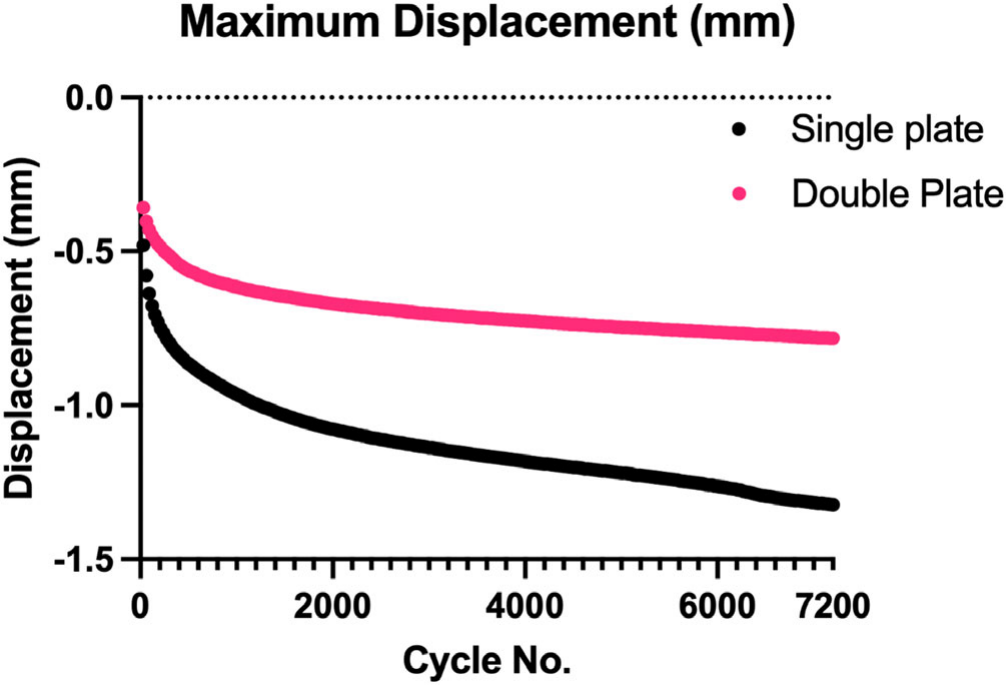
Maximum displacement (mm) of all single and double plated scapula over 7200 cycles.

**FIGURE 3:**
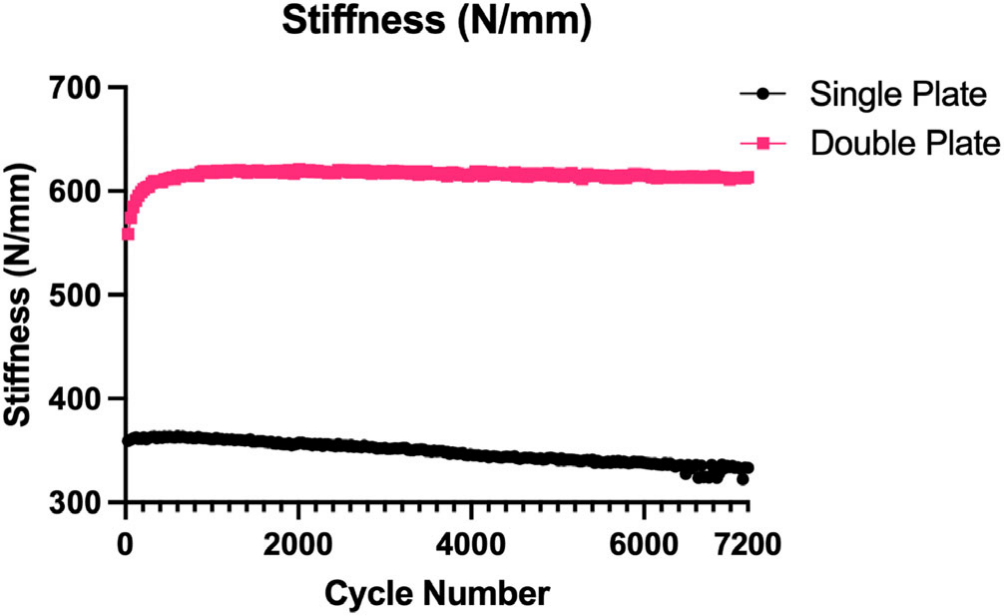
Stiffness (N/mm) of all single and double plated scapula over 7200 cycles.

Overall mean displacement was −0.6566 mm (± 0.4486 mm) with skewness of 1.5737 and kurtosis 2.2999, maximum displacement –0.8483 (± 0.49632) with skewness –1.4560 and kurtosis 1.6056, and mean stiffness of 471.2 N (± 178.123 N) with skewness −0.0079 and kurtosis –1.1717. Variability due to a dog effect (intraclass correlation coefficient) when adjusted for treatment (single vs. double plate) was low with a range of 3% to 6%. The mean displacement was significantly higher across all test cycles in the single plate group (Figure 1), with a predicted mean displacement of –0.81 mm compared to −0.48 mm for the double plate group (*p* < .0001). The difference between the predicted means was –0.33 mm (± 0.13 mm). Maximum displacement was significantly higher in the single plate (Figure 2) group with a predicted mean maximum displacement of −1.03 mm compared to –0.64 mm for the double plate group, (*p* < .0001). The difference between the predicted means was 0.39 mm (± 0.144 mm). Mean stiffness was significantly lower in the single plate group with a predicted mean of 392.8 N/mm compared to 563.7 N/mm for the double plate group (*p* < .0001) (Figure 3). The difference between the predicted means was 170.9 N/mm (65.06 N).

**FIGURE 4:**
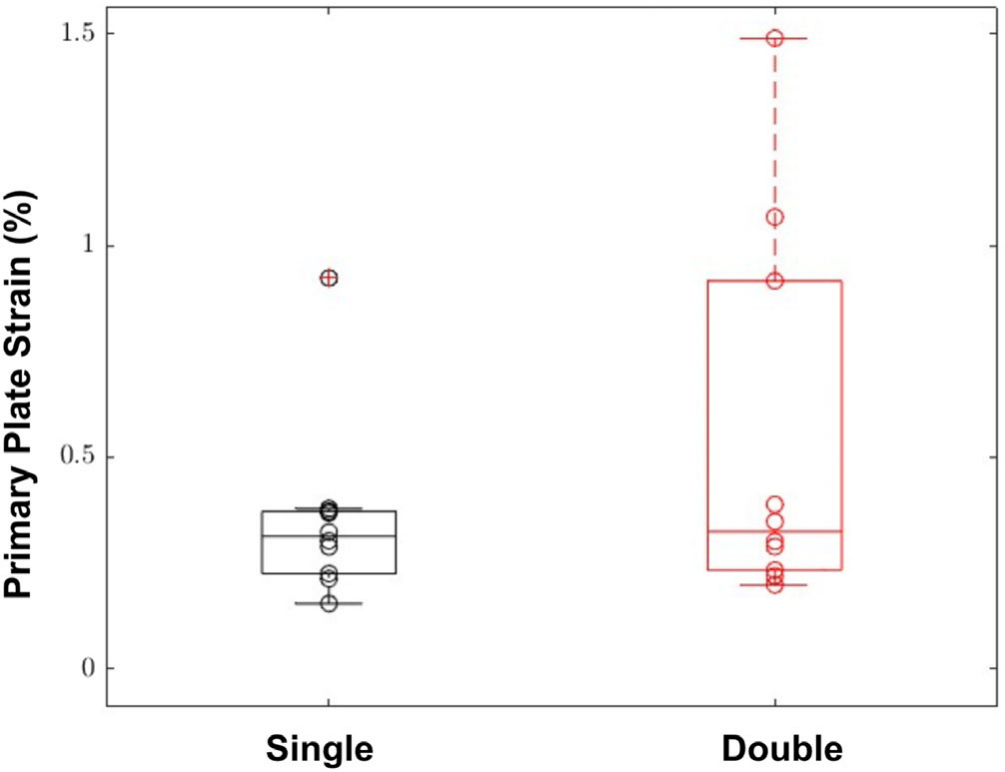
Primary plate strain (%) of both single and double plated scapula at maximum displacement.

Of the 24 scapulae successfully tested and included in statistical analysis, 20 were available for analysis of plate strain using 3D DIC. Recording of the 3D DIC ended prematurely for four of the specimens, two in each group. At maximum displacement, the mean tensile strain of the primary plate in the region of the fracture gap was 0.44% (±0.21%), which was not statistically different to the mean strain of 0.53% (±0.45%) for the double plate group (Figure 4).

## 4 DISCUSSION

We partially accepted our hypothesis; the secondary plate did increase construct stiffness and reduced construct displacement during loading compared to single plate fixation. We rejected the second part of our hypothesis because there was no statistical difference in primary plate strain over the fracture gap at maximum displacement.

The position of the second plate in a double plate construct affects the biomechanical behavior of that construct.^15^ Parallel plating of the distal humerus in human orthopedics gives greater axial stiffness, strength and torsional stiffness than orthogonal plating.^16^ In the previous report by Mair et al. on double plate fixation of the scapula, the secondary plate was placed near the first, both bordering the scapula spine. They did not find a difference in displacement or stiffness between the single and double plate groups. In our study, the second plate was placed as caudally as possible on the scapula, maximizing the distance from the primary plate, increasing the moment arm of the secondary plate. Due to the flat shape of the scapula and the angle of the glenoid, during loading the caudal aspect of the scapula has some cranial to caudal motion. This is resisted by the width of the plate, which has a higher area moment of inertia than the thickness of the plate, further contributing to the increase in stiffness and lower displacement even though the plate itself was a smaller size than the primary plate.

There was a small dog effect, 6% or less, on how the specimens biomechanically behaved, supporting the importance of using matched pairs in this study. Despite using the same breed for all scapulae, there was some mild variation in morphology between individual dogs and this likely contributed to alterations in their biomechanical behavior. Performing the two‐way ANOVA allowed us to account for this variability in our statistical analysis.

Our 3D digital image correlation results did not show any difference in plate surface strain and showed a large amount of variability in plate surface strain between the single‐plate or double plate constructs (Figure 4). It is unknown why three of the samples in the double plate group had much higher strain than the other samples. This could be due to variability in individual bone morphometry or due to the unreliability of digital image correlation when using it on a bone rather than on a synthetic cylinder as has been previously reported.^17^ We expected primary plate strain to be reduced in the double plate group due to the findings by de Bruyn et al.^18^ where they found an orthogonal plate reduced primary plate strain. The lack of difference could be due to the difference in biomechanical testing. In the previous study they used four point bending and torsion compared to our study with a single force‐displacement and further testing should be considered to investigate the effect of a second plate on primary plate strain.^18^ The lack of a statistical difference in the plate strain data may also be secondary to the small number of constructs available for strain analysis. Due to the lower power of this test, the potential for type II error cannot be ruled out.

**FIGURE 5:**
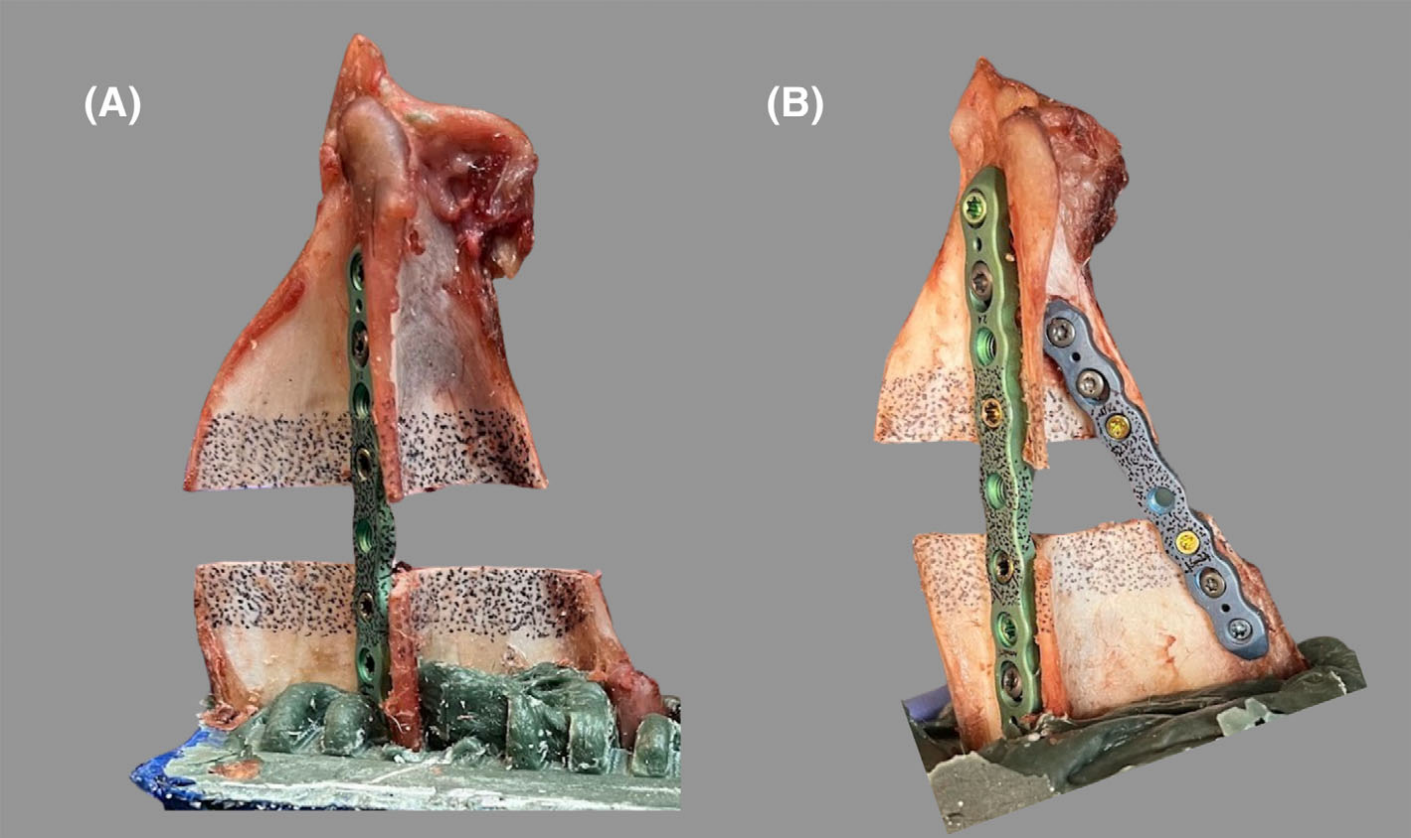
(A) A single plate specimen where the ostectomy remains parallel. (B) A double plate specimen where the addition of the caudal plate caused mild proximal displacement at the caudal aspect of the distal segment.

The decision to orient the scapula 20° from the vertical plane was replicated from the study done by Mair et al., who selected that angle based on standing angle measurements of five randomly selected large‐breed dogs. In this study, to better replicate a real clinical scenario and to decrease the risk of the actuator slipping, a 3D‐printed custom head, mimicking the humeral head was developed. While for most of the constructs, no slipping was noted, there were three cases that slipped during testing. All three cases that slipped were in the double plate group. The reason for slippage may have been because of variability in the potting angle. A custom‐made jig was used to reduce variability in potting angle, but despite this there may have been slight variations leading to a change in angulation of the glenoid predisposing to slippage. Since all three cases of slippage were in the double plate group the application of the caudal plate may have predisposed to slippage. When placing the secondary plate, the caudal aspect of the distal bone segment would be stabilized in a slightly more proximal position than in the single plate constructs where the caudal edge would tend to rotate slightly distally (Figure 5). This rotation was mild, <2 mm in all cases, and the clinical significance is unknown. The rotation caused slight sloping of the glenoid caudally, likely predisposing these specimens to slippage compared to the single plate group. Placing a wire mesh between the actuator and the articular surface of the glenoid cavity may have prevented actuator slippage.^5^

In this study, cyclic testing was chosen instead of testing single load to failure to represent the clinical scenario. In a similar study by Mair et al., their single plated constructs failed at a mean load of 3238 N and double plated constructs failed at a mean load of 3899 N. These failure loads are approximately 16 and 19 times more load, respectively, than the load applied here. The mechanism of construct failure was screw pull out through the thin scapula bone. Since our specimen set up was very similar, we predicted that construct failure would also occur through screw pull out and so we elected not to pursue load to failure testing.

For cyclic testing, the number of cycles and the load applied was chosen to represent 60 minutes of walking for a 15 kg dog. In both cases strain across the gap was calculated at less than 10% which is typically the level below which fracture healing is expected to occur in a comminuted fracture.^17^ The single plate constructs had a mean strain of 6.85% across the fracture gap compared to 4.27% for the double plate group. Although no guidelines currently exist, immediate postoperative weightbearing on a single plate may result in excess motion at the fracture site, which could result in implant failure and prevent bone healing. The lower strain across the fracture gap and higher stiffness of the double plate constructs supports using double plates for comminuted scapula fractures to eliminate the requirement for postoperative limb immobilization and allow for immediate postoperative weightbearing. Earlier return to weightbearing allows improved muscular recovery, prevents potentially irreversible cartilage damage and avoids disuse osteoporosis.^19^, ^20^ Immediate postoperative weightbearing would also remove the potential for complications from the Velpeau sling or Spica splint, which have previously been advised for scapula fractures.^21^ We have published a single case report of a dog with a comminuted scapula fracture stabilized with double plate fixation that was able to return to controlled weight bearing within 24 hours of surgery and successfully achieved bony union.^22^

The present study was limited due to its cadaveric nature and therefore we could not account for the tensile forces from the triceps brachii muscle on the caudal aspect of the scapula, the teres minor distally and the rhomboideus proximally. Additionally, although attempts were made throughout testing to ensure that the samples remained moist, results from testing of the constructs could have been affected by cadaveric nature and poor bone quality. Another limitation of the study was that the testing protocol only simulated 1 hour of walking, so we were unable to accurately predict long term implant stiffness or displacement. Finally, most of the current literature regarding the use of DIC for plate strain has been documented in long bones, making data acquisition much easier to achieve. In this study, the presence of the spine on the scapula made it more challenging to obtain accurate strain calculations and required multiple cameras to collect.

This study supports the treatment of severe comminuted scapula fractures with double plate fixation, to increase construct stiffness such that it allows an earlier return to controlled weightbearing postoperatively reducing the need for postoperative external coaptation. Further reports of clinical patients could address whether the stiffness and strength of the second plate can reliably allow for immediate weightbearing without implant failure.

## AUTHOR CONTRIBUTIONS

Barrett FM, BVM&S, DACVS (Small Animal): Conceptualization of the study and study design, data collection, data analysis and interpretation, drafting and revising manuscript, approval for the final article. Roth JD, PhD: Assistance in mechanical testing design, data collection, data analysis, drafting and revising manuscript, approval of the final article. Feller H, BS, MS: Data collection, data analysis and interpretation, drafting and revising the manuscript, approval of the final manuscript. McCarthy J, BVSc, DECVS, MRCVS: Conceptualization of the study and study design, data collection, data analysis and interpretation, drafting and revising manuscript, approval for the final article.

## CONFLICT OF INTEREST STATEMENT

This research was funded by the University of Wisconsin School of Veterinary Medicine Companion Animal Grant. Additional funding and implants were provided by Arthrex Inc. The other authors have no other conflicts of interest to disclose.
